# Potential therapeutic effects of curcumin, with or without L-DOPA, on motor and cognitive functions and hippocampal changes in rotenone-treated rats

**DOI:** 10.1007/s11011-025-01602-0

**Published:** 2025-04-10

**Authors:** Muaz Belviranlı, Nilsel Okudan, Tuğba Sezer

**Affiliations:** School of Medicine, Department of Physiology, Selçuk University, Konya, 42131 Turkey

**Keywords:** Parkinson's disease, Rotenone, Curcumin, L-DOPA, Hippocampus, Neurodegeneration

## Abstract

The neurodegenerative condition known as Parkinson’s disease (PD) is a long-term condition that causes both motor and non-motor symptoms. It is known that curcumin has a strong neuroprotective potential. This experimental study was designed to examine the anti-inflammatory, anti-apoptotic and neuroprotective effects of curcumin administered alone and in combination with L-DOPA in the hippocampus as well as behavioral symptoms in rotenone-induced PD model. Forty-two 4-month-old adult male Wistar rats were randomly divided into six groups as follows: Control, Curcumin, Rotenone, Rotenone plus curcumin, Rotenone plus L-DOPA and Rotenone plus curcumin plus L-DOPA. Control group received vehicles, curcumin group received curcumin (200 mg kg^−1^, daily for 35 days), rotenone group received rotenone (2 mg kg^−1^, daily for 35 days), and test groups received curcumin or L-DOPA (10 mg kg^−1^, daily for the last 15 days) or their combination in addition the rotenone. Pole, sucrose preference, open field, elevated plus maze, and Morris water maze tests were performed after treatment. Molecular and biochemical analyses were performed in the hippocampus tissue and serum samples. Rotenone injection caused impairments in motor activity, depressive-like behavior, and learning and memory functions. Rotenone also increased the expressions of α-synuclein, caspase 3, NF-κB, and decreased the expressions of parkin and BDNF in the hippocampus. However, especially curcumin and L-DOPA combined treatment normalized all these impaired molecular and behavioral variables. In conclusion, curcumin may exert beneficial effects in treatment strategies for PD-related hippocampal effects, especially when added to L-DOPA therapy.

## Introduction

The second most prevalent age-related neurodegenerative condition is Parkinson’s disease (PD), a chronic, complex, and progressive disorder (Poewe et al. 2017). The primary signs and symptoms of PD include bradykinesia, stiffness, resting-state tremor, and loss of postural reflexes (Postuma et al. 2015). Two main pathological changes are observed in PD: degeneration of dopaminergic neurons in the substantia nigra pars compacta and the presence of Lewy bodies containing fibrous, insoluble α-synuclein aggregates in surviving neurons. (Mor et al. 2019). The main reasons that cause dopaminergic neuronal degeneration and α-synuclein aggregation in PD include mitochondrial dysfunction, apoptosis, neuroinflammation and oxidative stress (Dernie 2020; Thirugnanam and Santhakumar 2022).

While PD is traditionally thought of as a motor disorder, there are several non-motor side effects as well. Among these, hippocampal dysfunction and resulting cognitive decline may occur in the earliest stages of PD, which may progress to dementia in the later stages of the disease (Kehagia et al. 2010; Aarsland et al. 2021). Patients with PD have up to six times the incidence of cognitive impairment compared to the general population (Aarsland et al. 2021). Studies conducted on rodents have reported that long term potentiation (LTP) and thus hippocampal synaptic plasticity is impaired in the experimental PD model (Nemani et al. 2010; Belloso-Iguerategui et al. 2023). It has been demonstrated that a combination of compromised dopaminergic and glutamatergic projections from the ventral tegmental region to the hippocampus causes the hippocampal dysfunction seen in PD (Belloso-Iguerategui et al. 2023). A member of the neurotrophin family, brain-derived neurotrophic factor (BDNF) is essential for learning and memory functions (Notaras and van den Buuse 2020). It has been shown that hippocampal BDNF levels decrease in an experimental PD model in rodents and that this decrease is associated with cognitive impairment (Rafie et al. 2023).

Despite extensive research, no effective treatment has yet been found to prevent or terminate the neurodegeneration process associated with PD (Fabbri et al. 2018). However, some pharmacological compounds are used to alleviate the clinical symptoms of the disease. L- 3,4-dihydroxyphenylalanine (L-DOPA), or levodopa, is considered the gold standard in treatment of PD. L-DOPA is the direct precursor of dopamine. It is transported to the brain and decarboxylated to dopamine, resulting in symptomatic relief by increasing dopamine levels in the striatum (Katzenschlager and Lees 2002) Although this drug is effective in relieving symptoms, it cannot inhibit neurodegeneration (Fahn et al. 2004). This has led researchers to be interested in natural compounds that have neuroprotective properties and can be potentially used in PD prevention. In this context, in recent years, some neuroprotective agents such as epigallocatechin, quercetin, resveratrol, and curcumin have been shown to be emerging options for this purpose, and significant progress has been made in research (Rabiei et al. 2019; Singh et al. 2020).

Curcumin is an active polyphenolic compound found in the rhizomes turmeric of Curcuma longa (Prasad et al. 2014). Curcumin has anti-inflammatory, anti-oxidant, anti-depressant, anti-apoptotic and anti-carcinogenic properties (Hatcher et al. 2008). Since curcumin can cross the blood-brain barrier due to its lipophilic nature (Yang et al. 2005), it is considered a promising agent in the treatment of neurodegenerative diseases (Adami and Bottai 2022). Although curcumin is one of the widely used phytochemicals due to its beneficial effects on health, there are controversial findings regarding its safety level. The low bioavailability of curcumin makes it difficult to generalize the effects observed in in vitro studies. It has also been reported that higher or irregular doses of curcumin may cause DNA damage and ROS production, inactivate tumor suppressor protein, affect systemic iron metabolism, and inhibit drug metabolizing enzymes such as cytochrome P450, glutathione-S-transferase, and UDP-glucuronosyltransferase (Burgos-Morón et al. 2010; Prasanth et al. 2024). Acute toxicity studies have reported that the maximum recommended dose level of curcumin is 5,000 mg/kg in rodents and 8 g/day in humans, and that even at these doses it does not cause toxicity (Burgos-Morón et al. 2010; Aggarwal et al. 2016).

In experimental PD models, it has been reported that curcumin has a neuroprotective effect by restoring dopamine levels, protecting dopaminergic neurons from death, and preventing α-synuclein aggregation (Zbarsky et al. 2005; Rajeswari and Sabesan 2008; Rajeswari 2006, He et al. 2022; Khosravi et al. 2023; Rathore et al. 2023). In the rotenone-induced PD model, both curcumin alone and in combination with levodopa or rasagiline ameliorated motor and behavioral disorders by reducing oxidative stress and DNA damage (El-Shamarka et al. 2023). In addition, curcumin ameliorated mitochondrial and oxidative stress damage in the rotenone-induced PD model by upregulating the expression of antioxidant and autophagic transcription factor Nrf2 (Rathore et al., 2023). Curcumin has also been shown to reverse the inflammatory response induced by 1-methyl- 4-phenylpyridinium ion in brain astrocytes by inhibiting the expression of proinflammatory effectors such as toll like receptor (TLR) 4 and nuclear factor kappa B (NF-κB). This suggests that curcumin may exert therapeutic effects on PD via its anti-inflammatory and anti-oxidant properties in neuronal cells (Yu et al. 2016). Although limited scientific evidence exists, curcumin has been shown to improve cognitive functions, which are impaired as a result of PD, by activating survival-related signaling pathways such as BDNF, and by reducing inflammatory markers and caspase activity in the hippocampus (Yang et al. 2014; Darbinyan et al. 2017, 2022; Madiha and Haider 2019; Motawi et al. 2020).

In this study, an experimental PD model was established using rotenone. Rotenone is a neurotoxic pesticide that disrupts oxidative phosphorylation in mitochondria (Schuler and Casida 2001). It specifically destroys dopaminergic neurons of the nigrostriatal pathway (Cannon et al. 2009) and therefore, it accurately reflects many aspects of human PD pathology. This study was designed to demonstrate the effect of curcumin supplementation with or without L-DOPA on the etiology of PD. Therefore, motor and cognitive functions were evaluated using Pole, sucrose preference, open field, elevated plus maze, and Morris water maze tests. Serum tumor necrosis factor- α (TNF-α) and interleukin (IL)− 1β levels were analyzed as markers of systemic inflammation. Additionally, expressions of α-synuclein, as a marker of PD pathology, BDNF, as a neurotrophic factor, parkin, as a marker of mitophagy, caspase 3, as a marker of apoptosis and NF-κB, as a marker of inflammation were measured in the hippocampus.

## Methods

### Chemicals and reagents

Rotenone was obtained from Bostonchem (Cat no: BRM- 817234, Boston, USA) and dissolved in sunflower oil. Curcumin and sucrose were purchased from Bio Basic Inc. (Cat no: CB0346 and SB0498, respectively, Ontario, Canada) and curcumin was dissolved in corn oil. Carbidopa/Levodopa was obtained from İlko İlaç San. Tic. AŞ (Dopadex SR 25/100, Turkey) and dissolved in physiological saline. TNF-α (Cat no: E0764Ra) and IL- 1β (Cat no: E0119Ra) ELISA kits were purchased from BT LAB (Bioassay Technology Laboratory, Shanghai, China).

### Animals

For this study, forty-two 4-month-old adult male Wistar rats weighing 450–550 g at the beginning of the experiment were obtained from Experimental Medicine Research Center of Selçuk University. Animals were kept in temperature (23 ± 1 ^o^C), humidity (50%) and light (12/12 light/dark) controlled rooms throughout the study. Standard rat chow and water were given *ad libitum*. The research protocol was approved by the local ethics committee (Approval code: 2021 - 40). All experimental procedures were performed in accordance with ethical standards established by the National Institutes of Health.

### Experimental design and treatment regimen

Before starting the experiment, the animals were randomly divided into six groups as follows:

1. Control (Con; *n* = 6): The animals were given the vehicles of rotenone (sunflower oil), curcumin (corn oil) and L-DOPA (physiological saline) for the same duration, amount and route for 35 days.
2. Curcumin (Cur; *n* = 6): The animals were administered 200 mg kg− 1 curcumin via oral gavage for 35 days, and the vehicles of rotenone and L-DOPA were also administered for the same duration, amount and route.
3. Rotenone (Rot; *n* = 7): The animals were subcutaneously injected with 2 mg kg− 1 rotenone for 35 days, and the vehicles of curcumin and L-DOPA were also administered for the same duration, amount and route.
4. Rotenone plus curcumin (Rot + Cur; *n* = 7): The animals were injected subcutaneously with 2 mg kg− 1 rotenone for 35 days, and 200 mg kg− 1 curcumin was given via oral gavage for the last 15 days.
5. Rotenone plus L-DOPA (Rot + L-DOPA; *n* = 7): The animals were injected subcutaneously with 2 mg kg− 1 rotenone for 35 days, and 10 mg kg− 1 L-DOPA was administered via oral gavage for the last 15 days.
6. Rotenone plus curcumin plus L-DOPA (Rot + Cur + L-DOPA; *n* = 7): The animals were injected subcutaneously with 2 mg kg− 1 rotenone for 35 days, and 200 mg kg− 1 curcumin and 10 mg kg− 1 L-DOPA were administered via oral gavage for the last 15 days.

**Fig. 1:**
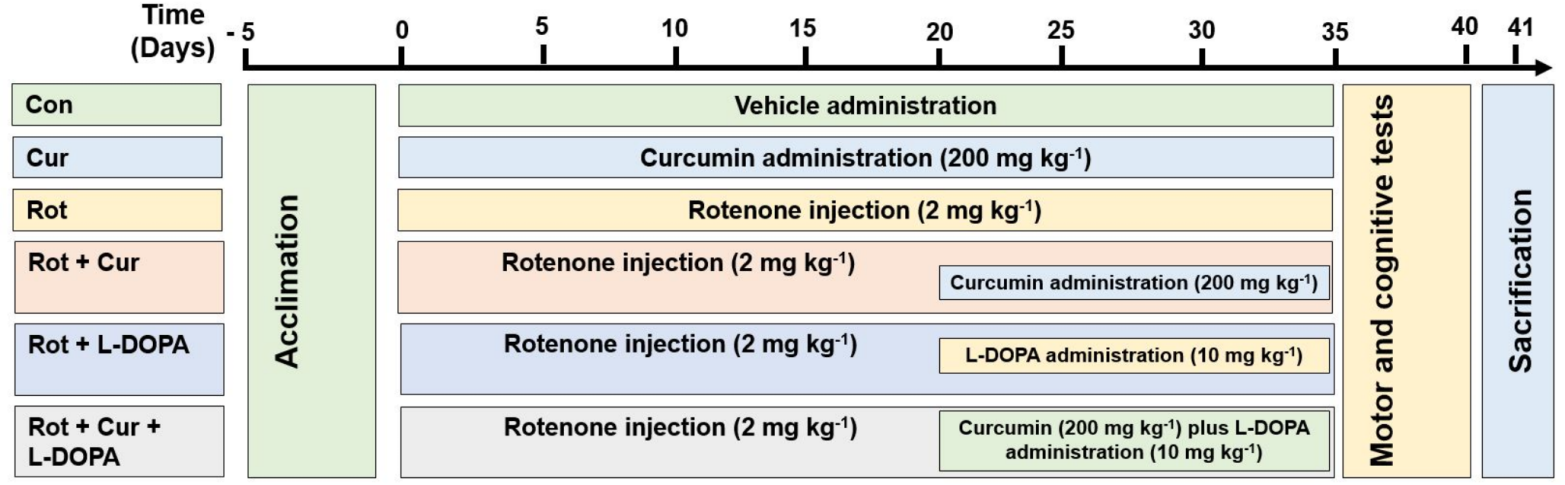
Diagrammatic representation of the timetable and methodology for the experiment

Since 2 rats died due to unexpected reasons at the beginning of the study, the control and curcumin groups consisted of 6 rats, and the other groups consisted of 7 rats each. All solutions were freshly prepared. The study design and doses of rotenone (Thakur and Nehru 2013; Palle and Neerati 2018), curcumin (Darbinyan et al. 2017)d DOPA (Alam and Schmidt 2004) were selected based on previous studies. In this study, since the total duration of the study in the groups where the therapeutic effect of curcumin and/or L-DOPA was examined was 35 days, a total of 35-day treatment protocol was applied to the groups given alone curcumin, rotenone and vehicle. After the 35-day treatment protocol, the groups’ motor and behavioral functions were evaluated with various tests. Figure 1 displayed the current experiment’s timeframe and experimental design.

### Assessment of motor and behavioral functions

#### Pole test

The Pole test is widely used to examine movement disorders associated with the basal ganglia, specifically bradykinesia, in rodents. The test was briefly performed as follows: Rats were placed on a vertical wooden pole with a diameter of 2.5 cm and a height of 100 cm, placed in a standard cage. The time it took for rats to descend from the top of the pole to the ground was recorded (Yanpallewar et al. 2004). The test was repeated 3 times with 1 min intervals and the average of the 3 tests was taken. The longer the rat stays on the pole and the longer it takes to get off the pole, it is considered to have good motor control.

#### Sucrose preference test

The sucrose preference test is used to measure anhedonia in rodents, which is expressed as a decreased ability to experience pleasure, a fundamental symptom of depression (Mazarati et al. 2007). During the 24-h training phase of the test, rats were given access to two pre-weighed bottles, one containing normal drinking water and the other containing 1% w/v sucrose solution. The next day, the bottles were refilled and their positions were changed. The bottles were weighed again after 48 h and the amount of sucrose and water consumed was calculated. Sucrose preference ratio (%) was calculated according to Bekris et al. (2005). Decreased sucrose preference was considered a sign of anhedonia (Zhao et al. 2021).

#### Open field test

The open field test is a popular method for evaluating rodent locomotor activity. A camera monitoring system was put on top of a wooden black box measuring 80 × 80 × 40 cm, which served as the experimental setting. The rats were released into the center of the open field and allowed to roam freely for 5 min. A computerized video tracking system was used to record each experiment, and the Ethovision XT 9.0 system (Noldus Information Technology, Wageningen, The Netherlands) was used for analysis. Before placing the next animal, the arena was cleaned with 70% ethanol in order to remove any potential bias resulting from odors left by the preceding animals. The number of rearings, total distance traveled (cm), time spent as a mobile (s), and speed (cm s^−1^) were measured (Belviranli & Okudan 2019).

#### Elevated plus maze

The elevated plus maze test is a widely used test for the evaluation of anxiety-like behaviors in rodents. The testing protocol was performed as previously described (Belviranli & Okudan 2019). Briefly, the test apparatus consisted of two open and two closed arms in the shape of a cross and the central region at their intersection. The apparatus was 50 cm above the ground. Rats were placed in the center and allowed to explore freely for 5 min. Animal movements were tracked with a camera and tracking software (Ethovision XT 9.0, Noldus Information Technology, Wageningen, The Netherlands). The anxiety index was calculated according to Cohen et al. (2013). Between sessions, the arena was cleaned with 70% ethanol solution.

#### Morris water maze test

The Morris water maze test is a widely used test to detect cognitive functions in rodents. In the Morris water maze test, a pool with a diameter of 150 cm, a height of 60 cm and a water temperature of 22–25 °C was used. The test consists of two parts: navigation and spatial probe tests. During the navigation test, the maze was divided into four imaginary quadrants, and the escape platform was placed in target quadrant, approximately 1 cm under water. During the four-day test, rats were randomly placed in four quadrants of the water maze each day. The rats were monitored for 1 min, and if they could not find the platform on their own at the end of this period, they were directed to the platform and allowed to stay on the platform for 30 s. The time it took the rats to find the hidden platform and the total distance moved were used as indicators of learning capacity. On the fifth day, a spatial probe test for spatial memory function was performed. Briefly, the platform was removed and the rats were allowed to roam freely in the pool for 90 s. Then, parameters such as the time spent in the target quadrant, the number of platform zone crossings and the time spent there, and the total distance traveled were recorded and analyzed using a software (Ethovision XT 9.0, Noldus Information Technology, Wageningen, The Netherlands) (Belviranli & Okudan 2019).

### Euthanasia and sample collection

After the behavioral tests were completed, the rats were anesthetized with ketamine/xylazine (60/10 mg kg^−1^, i.p.) and whole blood was collected from their hearts. Samples were transferred to non-additive tubes, centrifuged at 2000 g for 10 min, and serum samples were separated. Then the animals were sacrificed by cervical dislocation. Brain tissues were immediately removed and the hippocampus was isolated on ice. All samples were stored at − 80 ^o^C until time of analysis.

### Biochemical assay

Serum IL- 1β and TNF-α levels were determined using rat-specific commercially available ELISA kits. All measurements were performed in accordance with the manufacturer’s instructions. Briefly, 50 µL of standard diluted 1:2 to 1:16 was added to the standard wells, 40 µL of sample and 10 µL of anti-Bcl2 antibody were added to the sample wells, and finally, 50 µL of streptavidin-HRP was added to the sample and standard wells. The plate was sealed and incubated at 37 °C in the dark for one hour. Then, the plate was washed 3 times with 300 µM wash buffer. 50 µL of substrate solution A and substrate solution B were added to each well. The plate was sealed again and incubated at 37 °C in the dark for 10 min. Finally, 50 µL of stop solution was added and the absorbance value of each well was measured using a microplate reader with a wavelength of 450 nm.

### Quantitative real‑time polymerase chain reaction (qRT‑PCR) assay

Hippocampal gene expressions of α-synuclein, BDNF, parkin, caspase 3, and NF-κB were measured using specific primers and the housekeeping gene GAPDH. First, total RNA was isolated using RNA isolation reagent (Ribozol, #N580, VWR International, USA) according to the manufacturer’s instructions. Total RNA concentration and purity (260/280 absorbance ratio) were confirmed. Complementary DNA (cDNA) was then prepared using the commercially available kits (#1708891, Bio-Rad Laboratories, CA, USA). Table 1 shows the primer sequences of the analyzed genes. SYBR Green qPCR Master Mix (#1708880, iQ SYBR Green Supermix, Bio-Rad Laboratories, CA, USA) was used to determine the expression levels of target genes in the hippocampus tissues of the groups with the CFX- 96 Real-Time PCR System (Bio-Rad Laboratories, CA, USA). The Ct values were used to calculate the relative expression by the 2^−ΔΔCt^ method (Schmittgen and Livak 2008), setting the control as 1.0.

**Table 1:** Forward and reverse primers of genes used for qRT-PCR experiments

Gene	Primer sequence
Parkin	Forward: CTCAGACAAGGACACATCAGTAG Reverse: TACATTGGAAGACCAAGACAGG
α-synuclein	Forward: CCTAGCAGTGAGGCTTATGAAA Reverse: GAACACCTGGGCAGATCTTAG
BDNF	Forward: TCATACTTCGGTTGCATGAAGG Reverse: ACACCTGGGTAGGCCAAGTT
caspase 3	Forward: CCACGGAATTTGAGTCCTTCT Reverse: CCACTCCCAGTCATTCCTTTAG
NF-κB	Forward: GGTTACGGGAGATGTGAAGATG Reverse: GTGGATGATGGCTAAGTGTAGG
GAPDH	Forward: ACTCCCATTCTTCCACCTTTG Reverse: CCCTGTTGCTGTAGCCATATT

### Statistical analysis

The statistical evaluations were carried out with GraphPad Prism 9.0 (GraphPad Software Inc., San Diego, CA, USA). Normality of data distributions was checked with the Kolmogorov-Smirnov test. Normally distributed data sets were analyzed using one-way ANOVA followed by Tukey post-hoc test with Bonferroni correction. For data sets that did not comply with normal distribution, the Kruskal-Wallis test was applied followed by the post-hoc Dunn test. Total distance traveled and latency during the MWM navigation test were analyzed using a repeated-measures two-way ANOVA test. The data are represented as the mean ± standard deviation (SD) and statistically significant differences were set at *P* < 0.05.

## Results

### Effect of curcumin with or without L-DOPA on motor function, and depression and anxiety level

**Fig. 2:**
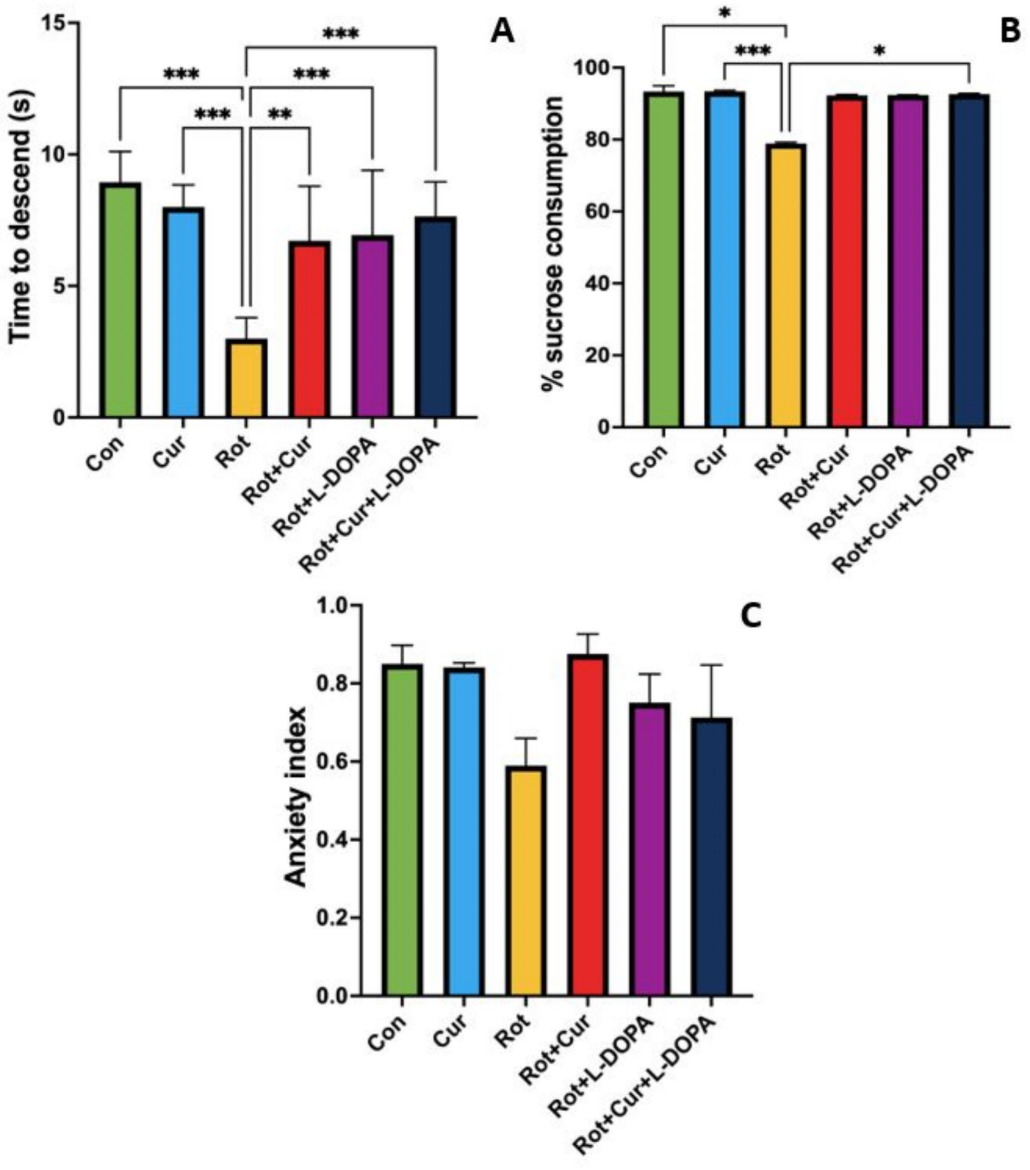
Effects of curcumin and/or L-DOPA on (**a**) motor function, (**b**) depressive-like behavior and (**c**) anxiety index. Values are expressed as the mean ± SD. ^*^P < 0.05; ^**^P < 0.01, ^***^P < 0.001. Statistical significance was determined by one-way ANOVA (a) and Kruskal-Wallis (b and c) tests. Con: Control group (*n* = 6); Cur: Curcumin group (*n* = 6); Rot: Rotenone group (*n* = 7); Rot + Cur: Rotenone plus curcumin group (*n* = 7); Rot + L-DOPA: Rotenone plus L-DOPA group (*n* = 7); Rot + Cur + L-DOPA: Rotenone plus curcumin plus L-DOPA group (*n* = 7)

In this study, motor function was evaluated with the Pole test, depressive-like behavior was evaluated with the sucrose preference test, and anxiety level was evaluated with the anxiety index calculated in the EPM test. In the pole test, the descend time was lower in the Rot group than in all other groups (F_(5,34)_ = 11.1, *P* < 0.001, Fig. 2a). In the sucrose preference test, the % sucrose consumption was lower in the Rot group than in the Con, Cur and Rot + Cur + L-DOPA groups (KW = 21.74, *P* < 0.001, Fig. 2b). However, there was no statistically significant difference among the groups in the anxiety index (KW = 8.350, *P* = 0.14, Fig. 2c).

### Effect of curcumin with or without L-DOPA on locomotion

**Fig. 3:**
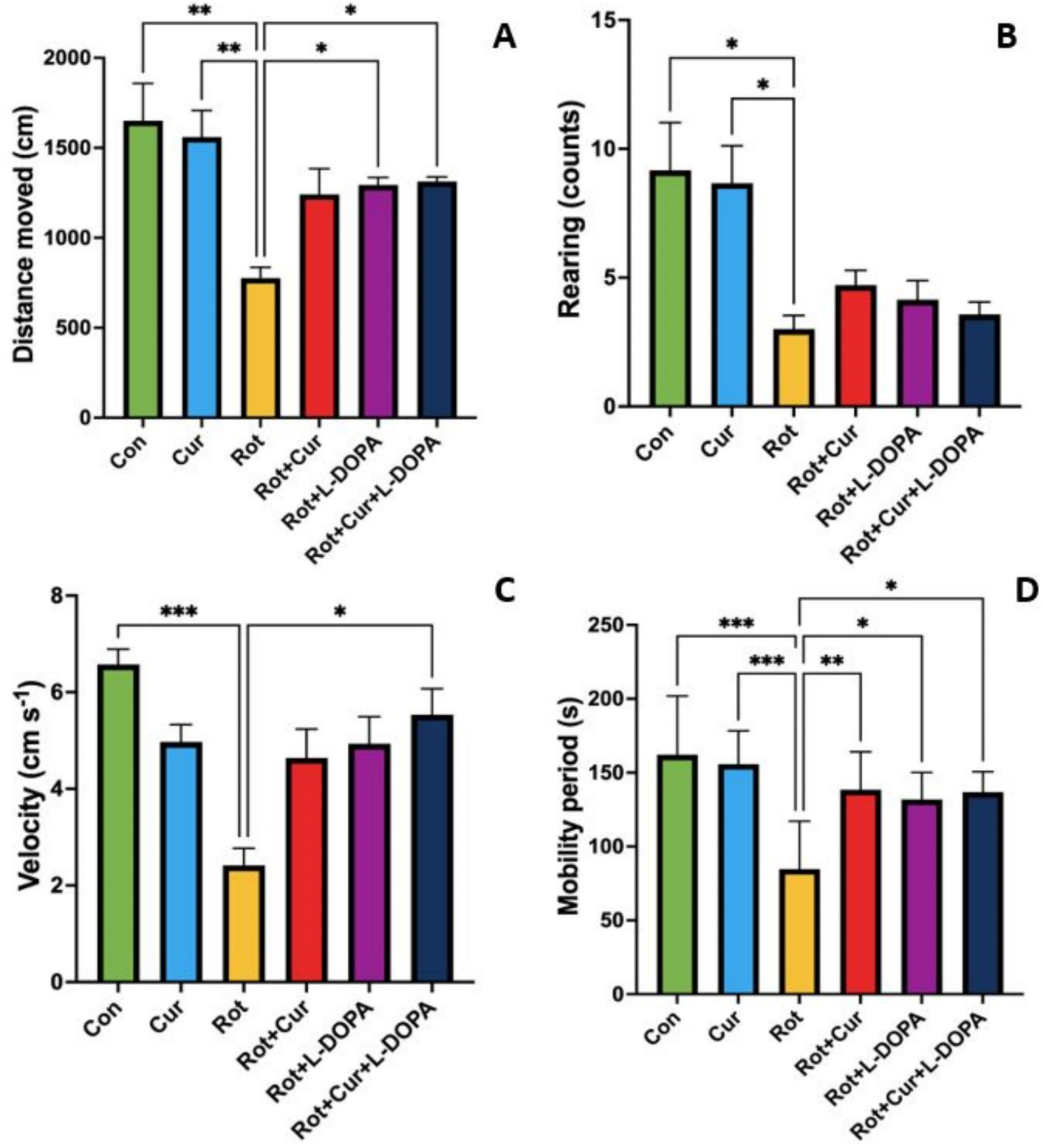
Effects of curcumin and/or L-DOPA on (**a**) total distance traveled, (**b**) rearing counts, (**c**) velocity and (**d**) time spent as a mobile during the open field test. Values are expressed as the mean ± SD. ^*^P < 0.05; ^**^P < 0.01, ^***^P < 0.001. Statistical significance was determined by one-way ANOVA (d) and Kruskal-Wallis (a, b and c) tests. Con: Control group (*n* = 6); Cur: Curcumin group (*n* = 6); Rot: Rotenone group (*n* = 7); Rot + Cur: Rotenone plus curcumin group (*n* = 7); Rot + L-DOPA: Rotenone plus L-DOPA group (*n* = 7); Rot + Cur + L-DOPA: Rotenone plus curcumin plus L-DOPA group (*n* = 7)

In this study, locomotion was evaluated using the open field test. The total distance traveled was lower in the Rot group than in the Con, Cur, Rot + L-DOPA and Rot + Cur + L-DOPA groups (KW = 19.52, *P* = 0.002, Fig. 3a). Rearing counts were lower in the Rot group than in the Con and Cur groups (KW = 19.25, *P* = 0.002, Fig. 3b). The average speed was lower in the Rot group than in the Con and Rot + Cur + L-DOPA groups (KW = 21.37, *P* < 0.001, Fig. 3c). Finally, the time spent as a mobile was lower in the Rot group than in all other groups (F_(5,34)_ = 7.07, *P* < 0.001, Fig. 3d).

### Effect of curcumin with or without L-DOPA on spatial learning and memory function

**Fig. 4:**
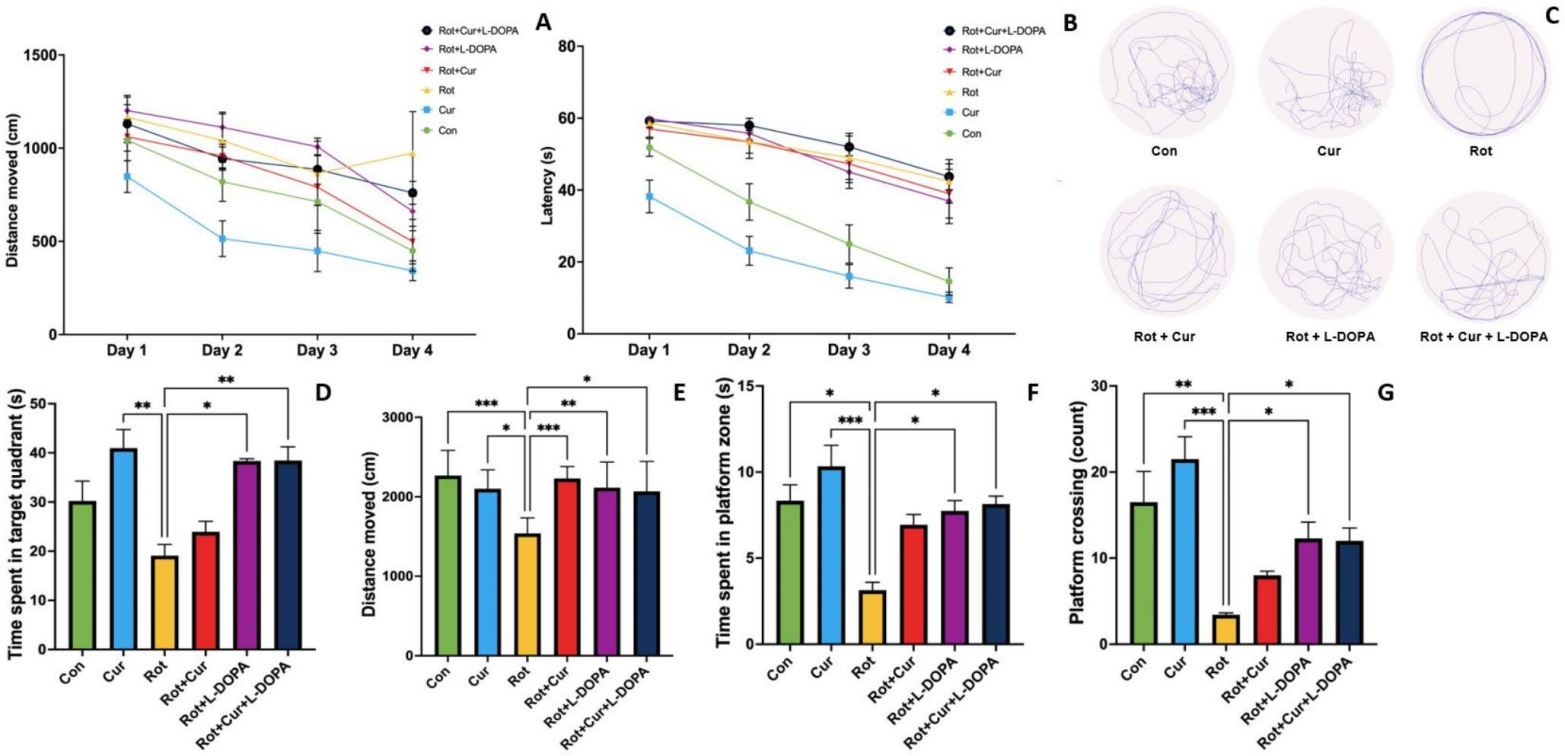
Effects of curcumin and/or L-DOPA on (**a**) total distance traveled and (**b**) latency to reach platform during the navigation test, (**c**) representative recorded track plot during spatial probe test, (**d**) time spent in target quadrant, (**e**) total distance traveled (**f**) time spent in platform zone and (**g**) platform crossing counts during the probe test session of the Morris Water Maze. Values are expressed as the mean ± SD. ^*^P < 0.05; ^**^P < 0.01, ^***^P < 0.001. Statistical significance was determined by repeated-measures two-way ANOVA (a and b), one-way ANOVA (e) and Kruskal-Wallis (c, d, f and g) tests. Con: Control group (*n* = 6); Cur: Curcumin group (*n* = 6); Rot: Rotenone group (*n* = 7); Rot + Cur: Rotenone plus curcumin group (*n* = 7); Rot + L-DOPA: Rotenone plus L-DOPA group (*n* = 7); Rot + Cur + L-DOPA: Rotenone plus curcumin plus L-DOPA group (*n* = 7)

In this study, the effect of rotenone injection on learning and memory functions and the possible effects of curcumin and/or L-DOPA administration on this process were evaluated using the Morris water maze test. During the 4-day navigation test period, the total distance traveled by all groups except the Rot group decreased over time (Con = F_(3,15)_ = 21.65, *P* < 0.001; Cur = F_(3,15)_ = 12.34, *P* < 0.001; Rot = F_(3,18)_ = 1.01, *P* = 0.370; Rot + Cur = F_(3,18)_ = 13.10, *P* = 0.003; Rot + L-DOPA = F_(3,18)_ = 33.49, *P* < 0.001; Rot + Cur + L-DOPA = F_(3,18)_ = 22.67, *P* < 0.001). The swimming distance was higher in the Rot group than in the Con and Cur groups on the second (F_(5,34)_ = 4.54, *P* = 0.003) and fourth (F_(5,34)_ = 3.39, *P* = 0.014) days of the MWM navigation test (Fig. 4a). Latency to find platform decreased with repeated tests in all groups (Con = F_(3,15)_ = 26.39, *P* < 0.001; Cur = F_(3,15)_ = 34.88, *P* < 0.001; Rot = F_(3,18)_ = 5.99, *P* = 0.018; Rot + Cur = F_(3,18)_ = 6.26, *P* = 0.025; Rot + L-DOPA = F_(3,18)_ = 8.89, *P* = 0.007; Rot + Cur + L-DOPA = F_(3,18)_ = 14.95, *P* = 0.002). Latency to find platform was higher in the Rot group than in the Cur group on all days of the MWM navigation test (Day 1, KW = 23.34, *P* < 0.001; Day 3, KW = 22.83, *P* < 0.001; Day 3, KW = 20.23, Day 4, KW = 20.30, *P* < 0.001) (Fig. 4b).

Spatial probe test was performed on day 5 to evaluate the spatial memory abilities of the groups. The time spent in the target quadrant was lower in the Rot group than in the Cur, Rot + L-DOPA and Rot + Cur + L-DOPA groups (KW = 23.91, *P* < 0.001, Fig. 4c and d). The total distance traveled was lower in the Rot group than in all other groups (F_(5,34)_ = 6.19, *P* < 0.001, Fig. 4e). The time spent in the platform zone and the number of platform crossing were lower in the Rot group than in the Con, Cur, Rot + L-DOPA and Rot + Cur + L-DOPA groups (KW = 28.11, *P* < 0.001, Fig. 4f and KW = 30.69, *P* < 0.001, Fig. 4g, respectively).

### Effect of curcumin with or without L-DOPA on circulating inflammatory markers

**Fig. 5:**
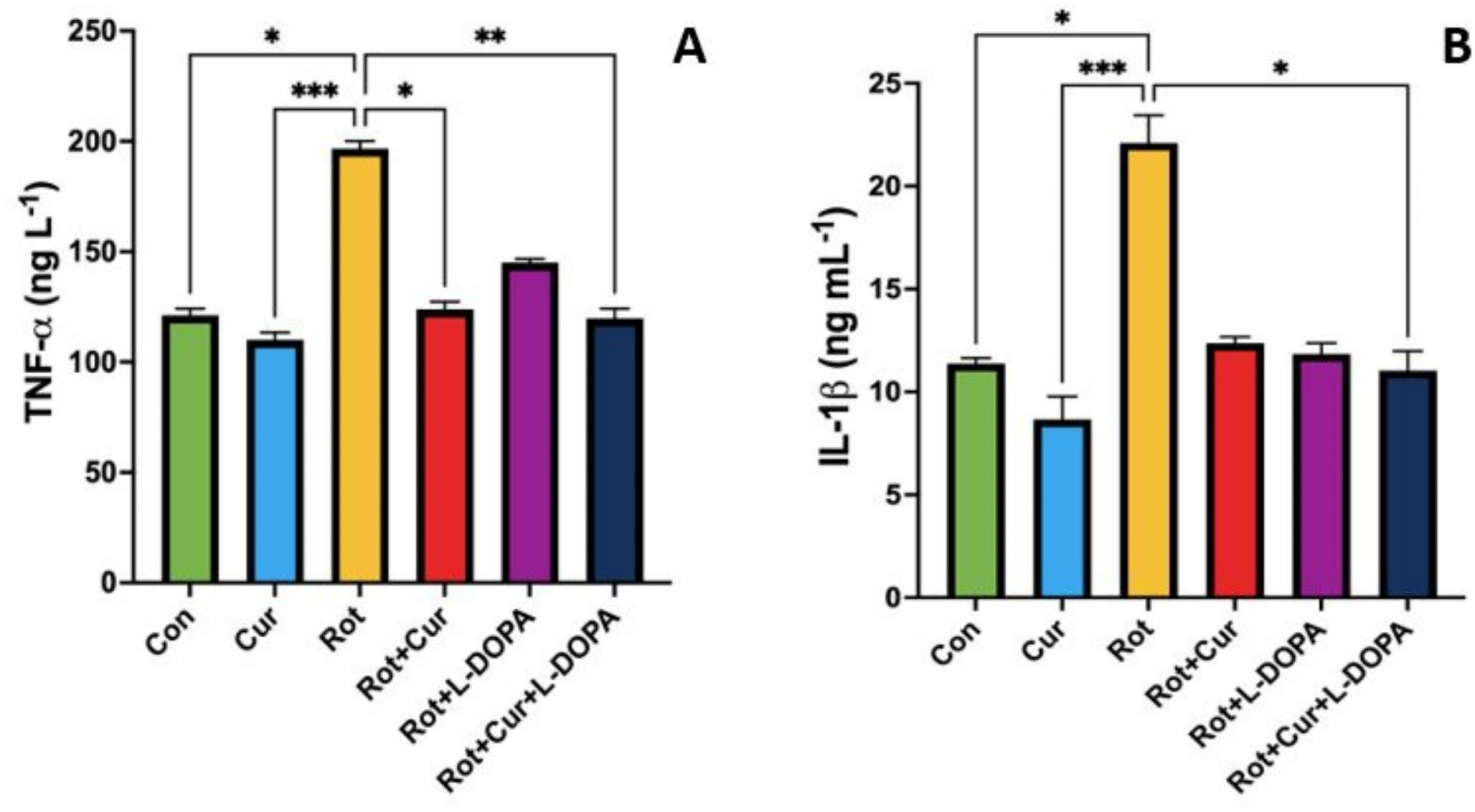
Effects of curcumin and/or L-DOPA on serum (**a**) TNF-α and (**b**) IL- 1β levels. Values are expressed as the mean ± SD. ^*^P < 0.05; ^**^P < 0.01, ^***^P < 0.001. Statistical significance was determined by the nonparametric independent-samples Kruskal-Wallis test. Con: Control group (*n* = 6); Cur: Curcumin group (*n* = 6); Rot: Rotenone group (*n* = 7); Rot + Cur: Rotenone plus curcumin group (*n* = 7); Rot + L-DOPA: Rotenone plus L-DOPA group (*n* = 7); Rot + Cur + L-DOPA: Rotenone plus curcumin plus L-DOPA group (*n* = 7)

In this study, the effect of rotenone injection on systemic inflammation and the possible effect of curcumin and/or L-DOPA administration on this process were determined by measuring TNF-α and IL- 1β levels in serum samples. Serum TNF-α level was higher in the Rot group than in the Con, Cur, Rot + Cur and Rot + Cur + L-DOPA groups (KW = 31.93, *P* < 0.001, Fig. 5a). Similarly, IL- 1β level was higher in the Rot group than in the Con, Cur and Rot + Cur + L-DOPA groups (KW = 22.49, *P* < 0.001, Fig. 5b).

### Effects of curcumin with or without L-DOPA on hippocampal gene expressions

**Fig. 6:**
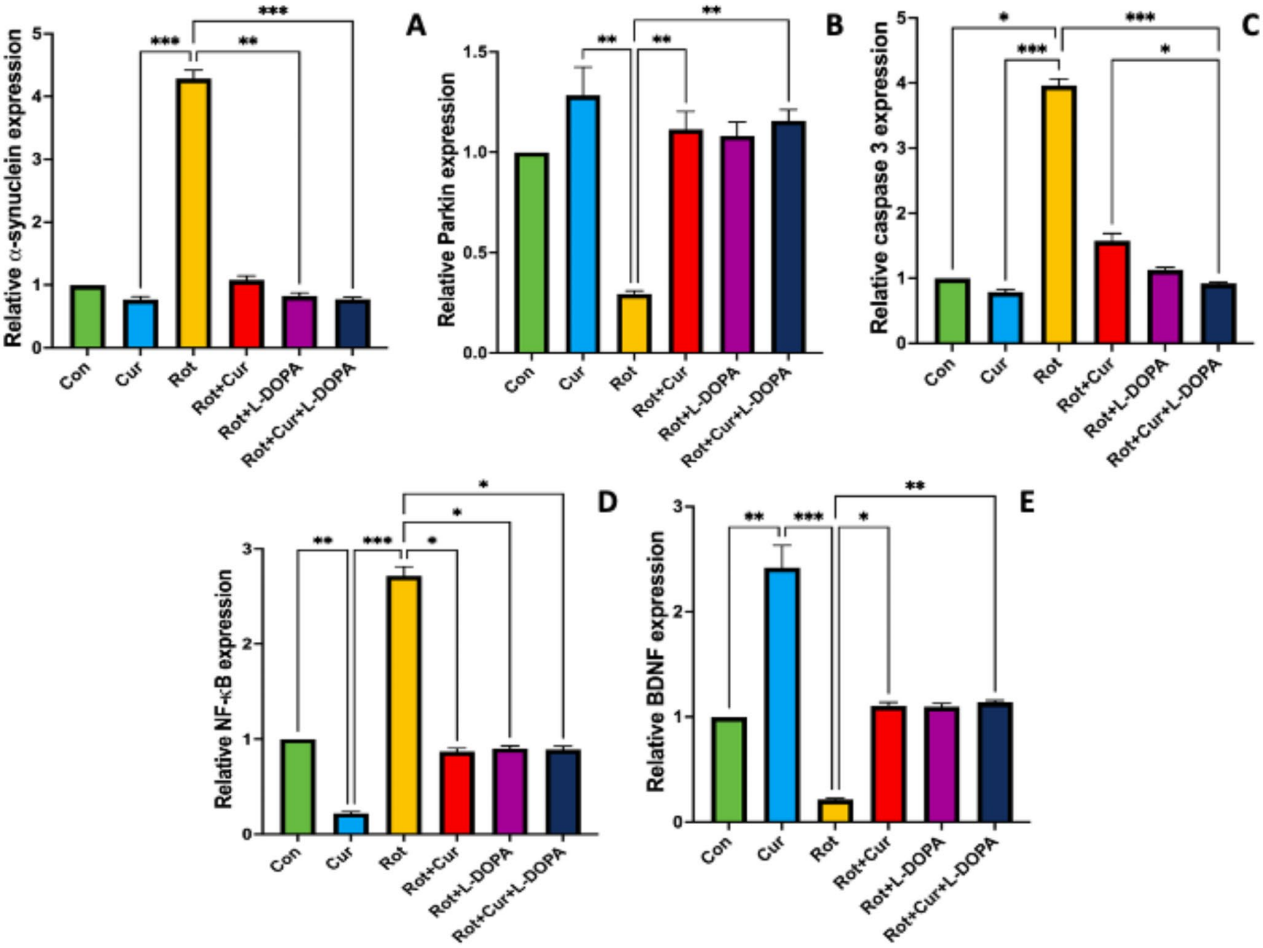
Effects of curcumin and/or L-DOPA on hippocampal expressions of (**a**) α-synuclein, (**b**) parkin, (**c**) caspase 3, (**d**) NF-κB and (**e**) BDNF. Values are expressed as the mean ± SD. ^*^P < 0.05; ^**^P < 0.01, ^***^P < 0.001. Statistical significance was determined by the nonparametric independent-samples Kruskal-Wallis test. Con: Control group (*n* = 6); Cur: Curcumin group (*n* = 6); Rot: Rotenone group (*n* = 7); Rot + Cur: Rotenone plus curcumin group (*n* = 7); Rot + L-DOPA: Rotenone plus L-DOPA group (*n* = 7); Rot + Cur + L-DOPA: Rotenone plus curcumin plus L-DOPA group (*n* = 7)

Expressions of α-synuclein, parkin, caspase 3, NF-κB and BDNF were examined in hippocampus tissue. α-synuclein expression was higher in the Rot group than in the Cur, Rot + L-DOPA and Rot + Cur + L-DOPA groups (KW = 31.02, *P* < 0.001, Fig. 6a). Parkin expression was lower in the Rot group than in the Cur, Rot + Cur and Rot + Cur + L-DOPA groups (KW = 20.86, *P* < 0.001, Fig. 6b). Caspase 3 expression was higher in the Rot group than in the Con, Cur and Rot + Cur + L-DOPA groups. Additionally, it was lower in the Rot + Cur + L-DOPA group than in the Rot + Cur group (KW = 37.80, *P* < 0.001, Fig. 6c). NF-κB expression was higher in the Rot group than in all other groups (KW = 31.40, *P* < 0.001, Fig. 6d). BDNF expression was lower in the Rot group than in the Cur, Rot + Cur and Rot + Cur + L-DOPA groups. Additionally, it was higher the Cur group than in the Con group (KW = 31.48, *P* < 0.001, Fig. 6e).

## Discussion

The findings of this study showed that rotenone administration caused impairment in motor functions, depressive-like behaviors, attenuation of locomotor activity, and cognitive impairments in rats. These motor and behavioral changes are supported by biochemical and molecular findings, such as systemic inflammation shown by increased TNF-α and IL- 1β levels in the blood, increased expression of α-synuclein, caspase 3, NF-κB and decreased parkin and BDNF expression in the hippocampus. In addition, current findings indicate that combined treatment of L-DOPA and curcumin ameliorates rotenone-induced impairment in motor, cognitive and other behavioral functions. This improvement was accompanied by decreased systemic inflammation and decreased expression of α-synuclein, caspase 3, NF-κB and increased parkin and BDNF expression. Today, a growing body of evidence suggests that preventing and slowing the progression of PD may be possible with curcumin treatment (Nguyen et al. 2018; Donadio et al. 2022; Fikry et al. 2022). Most studies conducted to date have examined the effects of curcumin pre-treatment or co-treatment in the experimental PD model (Phukan et al. 2023; Darbinyan et al. 2023; Rathore et al. 2023). However, in a few studies (Madiha and Haider 2019; Xu et al. 2023), the therapeutic effect of curcumin has been investigated after inducing an experimental PD model. Additionally, as far as we know, there is no study investigating the therapeutic potential of curcumin alone or in combination with L-DOPA, especially on hippocampal functions and related molecular biomarkers, so our study is the first on this subject.

Various chemicals such as rotenone, 1-methyl- 4-phenyl- 1,2,3,6-tetrahydropyridine (MPTP) and 6-hydroxydopamine (6-OHDA) have been widely used to mimic and elucidate the pathogenesis of PD in rodents (Thirugnanam and Santhakumar 2022). Rotenone is a neurotoxic pesticide and, due to its lipophilic properties, can easily cross the blood-brain barrier and affect the brain. It inhibits complex I and suppresses reduced nicotinamide adenine dinucleotide (NADH)-ubiquinone reductase activity and disrupts oxidative phosphorylation in mitochondria (Schuler and Casida 2001). Rotenone specifically destroys dopaminergic neurons of the nigrostriatal pathway (Cannon et al. 2009). The mechanism of action of rotenone includes activation of mitochondria-dependent apoptotic pathways, increased oxidative stress, protein aggregation, proteasome deficiency and neuroinflammation (Waldmeier and Tatton 2004; Madiha and Haider 2019; Betarbet et al. 2006).

Motor disorders are one of the first symptoms to appear in PD. In this study, we observed characteristic behavioral changes in rats exposed to rotenone, such as impairments in motor coordination and locomotor activity assessed by Pole and Open Field tests, respectively, and our findings confirmed previous studies (Madiha and Haider 2019; Haider et al. 2020; Ablat et al. 2022). However, curcumin and L-DOPA administered alone or in combination improved motor coordination by bringing the descent time closer to the control group. Additionally, an improvement in locomotor activity was observed, especially with the combined administration of curcumin and L-DOPA. Current studies have reported that curcumin supplementation either improves (Madiha and Haider 2019; Geng et al. 2022; Cai et al. 2023) or does not affect (Cui et al. 2016; Ramires Júnior et al. 2021) motor coordination and locomotor activity in the experimental PD model. These differences in findings may be due to differences in the dosage and duration of rotenone and curcumin, as well as the curcumin formulation used and experimental design.

Depression is one of the most frequently reported neuropsychiatric disorders in PD and its cause is complex (Miller et al. 2007). It has been associated with altered 5-HT level, which is further associated with dopamine deficiency (Mayeux 1990). The findings of our current study are consistent with previous studies showing that rotenone significantly reduces sucrose consumption in the sucrose preference test and induces anhedonia (inability to enjoy sucrose consumption) in rats, thereby causing depressive-like behavior (Santiago et al. 2010). While curcumin and L-DOPA post-treatment did not affect sucrose consumption when administered alone, the combined treatment caused an increase in sucrose consumption. It has previously been shown that curcumin alone may have beneficial effects on depression in PD (Gilhotra and Dhingra 2010; Madiha and Haider 2019). However, the lack of effect of curcumin alone in our study may be due to differences in the dose, duration and formulation of curcumin we used. The current study revealed the anti-depressant effect of curcumin and L-DOPA combination in rotenone-treated rats.

In this study, the anxiety index assessed by the elevated plus maze test was not affected by rotenone injection or any treatment regimen. It has been reported that neurochemical changes in PD may cause anxiety (Prediger et al. 2012). Despite studies showing that the rotenone-induced PD model causes anxiety in rodents (Rao et al. 2019), there are also studies claiming that it does not affect anxiety (Noseda et al. 2016, Nemutlu Samur et al. 2022). Our results showed that rotenone and/or curcumin and L-DOPA treatment did not cause anxiety-like behaviors. It has been shown that the results of anxiety tests are significantly affected by differences in animal species and that while rotenone is not anxiogenic in Sprague-Dawley rats, it is anxiogenic in Wistar rats (Rex et al. 2004).

Cognitive decline is very common in PD and impairment of hippocampal synaptic transmission and cognitive functions has been demonstrated through different experimental models induced by rotenone (Darbinyan et al. 2017), MTPT (Ho et al. 2014) and 6-OHDA (Chen et al. 2014). Rotenone intoxication has been shown to increase blue extravasation and fibrinogen accumulation in the rodent hippocampus (Guo et al. 2022). Similar to existing studies (Yang et al. 2014; Guo et al. 2022; Rafie et al. 2023), exposure to rotenone in this study led to learning and memory impairments. Dysfunctions in various neurotransmitters, including dopamine, serotonin and noradrenaline, are thought to contribute to the cognitive decline associated with PD (O’Callaghan and Lewis 2017). In addition, the cognitive impairment caused by rotenone exposure that we observed in this study may also be due to decreased hippocampal BDNF expression. BDNF is one of the most studied neurotrophic factors and is a key regulator of brain development, neuroplasticity, and learning and memory functions (Notaras and van den Buuse 2019). It has been shown that changes in BDNF levels may be one of the underlying mechanisms of many neurodegenerative diseases and learning and memory disorders (Miranda et al. 2019). Consistent with our findings, hippocampal BDNF expressions and levels were found to be lower in rodent PD models, which was associated with hippocampal functions impairment in both electrophysiologically and behaviorally (Darbinyan et al. 2017; Zhu et al. 2011; Alzoubi et al. 2018). In this study, the neuroprotective effect of curcumin and/or L-DOPA treatment was clearly observed during the spatial probe session of the MWM test through a significantly higher number of platform crossings, time spent in the platform zone, and time spent in the target quadrant. Ultimately, these abnormal changes were reversed with L-DOPA alone and the combination of curcumin and L-DOPA. In the present study, treatment with L-DOPA alone and the combination of curcumin and L-DOPA only improved spatial memory but not learning ability. Spatial memory has been shown to depend on the dorsal hippocampus but not on the ventral hippocampus (Fanselow and Dong 2010). This suggests that L-DOPA alone and the combination of curcumin and L-DOPA affected the dorsal part of the hippocampus rather than the entire hippocampus. The dorsal hippocampus receives input from the entorhinal cortex via the perforating pathway and communicates with brain structures such as the thalamus, mammillary complex, and cortex via neural pathways mediated by the fornix (Witter et al. 2017; Aggleton et al. 2022). These connections facilitate the role of the hippocampus in memory and spatial navigation. In addition, impaired motor abilities caused by rotenone treatment may have also affected swimming ability in the MWM test. This improvement in cognitive functions may have been mediated by increased hippocampal BDNF expression. It has been shown that BDNF has a protective effect against neurodegeneration and, consistent with our findings, curcumin treatment increases hippocampal BDNF levels, especially in the experimental PD model (Jin et al. 2022). However, our data are inconsistent with other studies showing that curcumin pre- or post-treatment alone can reverse PD-induced spatial learning and memory deficits (Yang et al. 2014; Song et al. 2016). This difference in findings may be due to differences in the dose, duration and formulation of curcumin.

In addition to impairments in motor functions, another symptom of PD is the accumulation of α-synuclein in the brain. α-Synuclein is a presynaptic protein neuropathologically linked to PD, and its abnormal soluble oligomeric structures called protofibrils mediate disruption of cellular homeostasis and synaptic function and neuronal death through effects on a variety of intracellular targets. Additionally, secreted α-synuclein exerts detrimental effects on neighboring cells, including the seeding of aggregation, contributing to the spread of disease (Stefanis 2012). Consistent with our findings, increased α-synuclein accumulation and hippocampal dysfunction were detected in PD patients and rodents as a result of rotenone injection (Feng et al. 2006; Hall et al. 2014; Adamowicz et al. 2017). However, in this study, L-DOPA alone and its combination with curcumin reduced hippocampal α-synuclein expression. These findings support the potential protective role of the combination of L-DOPA and curcumin in the rotenone-induced PD model.

In the PD, activation of the NF-κB-dependent p53 signaling pathway causes pro-inflammatory cytokine release, leading to progressive degeneration of dopaminergic neurons (Yan et al. 2014). Additionally, it has been shown that rotenone-mediated microglial activation causes nuclear translocation of NF-κB, resulting in dopaminergic degeneration and α-synuclein accumulation (Sherer et al. 2003). Inflammatory cytokines such as TNF-α and interleukin 1β are also important mediators of inflammation and have attracted great attention regarding neuroinflammatory processes in PD (Sawada et al. 2006). They cause neurotoxicity either directly through receptor binding to dopaminergic neurons or indirectly through glial cell activation and expression of inflammatory factors (Hirsch and Hunot 2009). The demonstration in our study that rotenone injection causes an increase in NF-κB expression in the hippocampus and an increase in TNF-α and interleukin- 1β levels in the blood is consistent with previous reports (Wang et al. 2002; Hirsch and Hunot 2009; Alabi et al. 2019). Additionally, it has been reported that inhibition of NF-κB expression prevents dopaminergic neuron loss in the experimental PD model (Ghosh et al. 2007). The results of our study showed that administration of curcumin and L-DOPA alone or in combination inhibited NF-κB expression in the hippocampus of rotenone-treated rats. In addition, serum TNF-α levels were reduced by the administration of curcumin alone and in combination with L-DOPA, and serum IL- 1β levels were reduced by the combination of curcumin and L-DOPA. Previous studies have also reported that curcumin and its different formulations inhibit NF-κB expression in specific brain regions and reduce the levels of inflammatory cytokines in the blood of patients with PD and an experimental PD model (Baj and Seth 2018; Wang et al. 2009). Therefore, the anti-inflammatory activity of curcumin may also help relieve motor and behavioral symptoms caused by PD.

It is known that cell death in PD is associated with apoptosis and mitophagy (Dodel et al. 1999; Ben Youssef et al. 2021; Pickles et al. 2018). In this context, caspase- 3 is the primary messenger of the mitochondria-dependent apoptotic pathway and Parkin is the primary messenger of mitophagy (Rupinder et al. 2007; Liu et al. 2019). Parkin also delays the release of cytochrome c by postponing the growth of mitochondria and the activation of caspase- 3 in ceramide-mediated cell death (Darios et al. 2003). In our study, we showed that, consistent with previous reports, rotenone administration increased caspase 3 expression and decreased parkin expression in the hippocampus (Dodel et al. 1999; Ben Youssef et al. 2021; Pickles et al. 2018). The protective effect of the curcumin and L-DOPA combination observed in rotenone-induced PD in the hippocampus may be related to the increase in parkin and the decrease in caspase- 3 activation. Consistent with our findings, existing studies have shown that curcumin stimulates parkin expression while inhibiting caspase 3 activation (Sharma and Nehru 2018; Rathore et al. 2023). According to our findings from this study, caspase- 3 activation and Parkin inhibition play an important role in hippocampal cell death in PD. Therefore, curcumin may be an interesting treatment targeting the inhibition of caspase- 3 and activation of Parkin.

Although we believe these findings have important implications for PD patients, we also acknowledge that this study has some limitations. First of all, the fact that the levels of monoamines such as dopamine, serotonin and noradrenaline were not measured due to technical problems is seen as an important deficiency. Second, molecular analyzes were limited to the hippocampus only. In order to see the widespread effect, it is necessary to perform these analyzes in different brain regions. The third limitation is the lack of lack of liver/kidney screenings to rule out compound effects. The fourth limitation is that, since the total study duration was 35 days, a total of 35 days of treatment protocol was applied to the groups given only curcumin, rotenone and vehicle, while 15 days of treatment was applied to the treatment groups. Although the 15-day treatment protocol was also shown to be sufficient, this difference in duration may have made it difficult to interpret the data. Finally, since the bioavailability of curcumin is low, the use of different curcumin formulations may be useful to see the protective effect more clearly. In our future work, we will focus on compensating for these shortcomings and conducting more comprehensive research to further confirm our results.

In conclusion, our findings from this study show that curcumin, especially when administered in combination with L-DOPA, improves motor and non-motor symptoms in a rotenone-induced PD rat model, and this is mediated by the reduction of neuroinflammation, the suppression of apoptotic pathways, and the stimulation of neurotrophic factors at the hippocampal level. Therefore, we think that curcumin may play a role in therapeutic strategies for hippocampal effects associated with PD.

## Data Availability

No datasets were generated or analysed during the current study.
